# Automatic segmentation and volume measurement of anterior visual pathway in brain 3D-T1WI using deep learning

**DOI:** 10.3389/fmed.2025.1530361

**Published:** 2025-04-28

**Authors:** Yongliang Han, Haixiang Wang, Qi Luo, Jingjie Wang, Chun Zeng, Qiao Zheng, Linquan Dai, Yiqiu Wei, Qiyuan Zhu, Wenlong Lin, Shaoguo Cui, Yongmei Li

**Affiliations:** ^1^Department of Radiology, The First Affiliated Hospital of Chongqing Medical University, Chongqing, China; ^2^College of Physics and Electronic Engineering, Chongqing Normal University, Chongqing, China; ^3^College of Computer and Information Science, Chongqing Normal University, Chongqing, China

**Keywords:** magnetic resonance imaging, optic nerve, deep learning, convolutional neural network, medical image processing

## Abstract

**Objective:**

Accurate anterior visual pathway (AVP) segmentation is vital for clinical applications, but manual delineation is time-consuming and resource-intensive. We aim to explore the feasibility of automatic AVP segmentation and volume measurement in brain T1-weighted imaging (T1WI) using the 3D UX-Net deep-learning model.

**Methods:**

Clinical data and brain 3D T1WI from 119 adults were retrospectively collected. Two radiologists annotated the AVP course in each participant’s images. The dataset was randomly divided into training (*n* = 89), validation (*n* = 15), and test sets (*n* = 15). A 3D UX-Net segmentation model was trained on the training data, with hyperparameters optimized using the validation set. Model accuracy was evaluated on the test set using Dice similarity coefficient (DSC), 95% Hausdorff distance (HD95), and average symmetric surface distance (ASSD). The 3D UX-Net’s performance was compared against 3D U-Net, Swin UNEt TRansformers (UNETR), UNETR++, and Swin Soft Mixture Transformer (Swin SMT). The AVP volume in the test set was calculated using the model’s effective voxel volume, with volume difference (VD) assessing measurement accuracy. The average AVP volume across all subjects was derived from 3D UX-Net’s automatic segmentation.

**Results:**

The 3D UX-Net achieved the highest DSC (0.893 ± 0.017), followed by Swin SMT (0.888 ± 0.018), 3D U-Net (0.875 ± 0.019), Swin UNETR (0.870 ± 0.017), and UNETR++ (0.861 ± 0.020). For surface distance metrics, 3D UX-Net demonstrated the lowest median ASSD (0.234 mm [0.188–0.273]). The VD of Swin SMT was significantly lower than that of 3D U-Net (*p* = 0.008), while no statistically significant differences were observed among other groups. All models exhibited identical HD95 (1 mm [1-1]). Automatic segmentation across all subjects yielded a mean AVP volume of 1446.78 ± 245.62 mm^3^, closely matching manual segmentations (VD = 0.068 ± 0.064). Significant sex-based volume differences were identified (*p* < 0.001), but no age correlation was observed.

**Conclusion:**

We provide normative values for the automatic MRI measurement of the AVP in adults. The 3D UX-Net model based on brain T1WI achieves high accuracy in segmenting and measuring the volume of the AVP.

## 1 Introduction

The anterior visual pathway (AVP), comprising the optic nerve, optic chiasm, and optic tract, transmits visual stimuli from the retina to the lateral geniculate nuclei (1). Various benign and malignant diseases can affect the AVP, causing acute swelling, chronic axonal loss, and atrophy (2). Accurate segmentation is crucial for clinical applications such as disease diagnosis, treatment planning, and monitoring disease progression. Successful image-guided surgery requires precise delineation of key neuro-logical and vascular structures during preoperative planning (3, 4). Modeling structural changes in the AVP throughout disease progression is important for characterizing neuropathic diseases (5). However, accurate segmentation and measurement of AVP are challenging due to their long pathway, the complexity of neighboring anatomical structures, and the high precision required for clinical use (6).

Manual delineation of AVP structures is time-consuming, resource-intensive, and prone to subjectivity. Automated quantification of AVP location and volumetrics would enable larger, more robust studies and potentially improve assessment accuracy compared to manual methods. Although significant progress has been made in AVP segmentation, traditional atlas-based methods vary in effectiveness for smaller structures like the optic nerve and chiasm, with Dice similarity coefficients (DSC) ranging from 0.39 to 0.78 (7, 8). Recently, convolutional neural networks (CNNs) have achieved significant advancements in MRI-based visual pathway segmentation. Mansoor et al. (6) proposed a deep learning approach that combines prior shape models with morphological features for AVP segmentation. Zhao et al. (9) improved segmentation performance by integrating a 3D fully convolutional network with a spatial probabilistic distribution map, effectively addressing issues of low contrast and blurred boundaries. Xie et al. (10) developed cranial nerves tract segmentation (CNTSeg), a multimodal deep learning network that accurately segments five major cranial nerve tracts—including CN II (optic nerve), CN III (oculomotor nerve), CN V (trigeminal nerve), and CN VII/VIII (facial and vestibulocochlear nerves)—by fusing T1-weighted images, fractional anisotropy (FA) images, and fiber orientation distribution function (fODF) peaks. However, due to the complexity near the optic chiasm and artifacts caused by magnetic susceptibility, AVP segmentation results remain unsatisfactory. Pravatà et al. (11) manually segmented high-resolution MRI data from 24 healthy subjects to obtain biometric indicators such as volume, length, cross-sectional area, and ellipticity of the AVP, establishing a foundation for standardized analysis. Nevertheless, manual segmentation is time-consuming and affects the reproducibility of results. The small sample size lacks age- and gender-specific standard data, and the ellipticity calculations may be overestimated. Further research is needed to refine automated segmentation techniques and develop more robust, standardized analysis methods for the AVP.

To enable broad application of the AVP segmentation model in clinical practice (non-GPU cluster environments), we selected the lightweight 3D UX-Net architecture (12) for automatic segmentation of the AVP in brain 3D- T1-weighted imaging (T1WI). As an optimized Swin Transformer variant, this volumetric convolutional neural network achieves computational efficiency through three key innovations: (a) replacement of multilayer perceptron (MLP) blocks with pointwise convolutions to reduce parameter count; (b) strategic minimization of normalization and activation layers to streamline processing; (c) implementation of large-kernel volumetric depthwise convolutions that expand the global receptive field while maintaining memory efficiency. These architectural modifications collectively enable robust 3D medical image segmentation with 34% fewer trainable parameters than standard Swin Transformer implementations (12, 13). To evaluate the segmentation performance of the 3D UX-Net model, we compared it with the 3D U-Net, Swin UNEt TRansformers (UNETR), UNETR++, and Swin Soft Mixture Transformer (Swin SMT) models (14–17). This comparative framework allows comprehensive evaluation of 3D UX-Net’s performance across model efficiency, anatomical detail preservation, and clinical applicability metrics. Our approach outperforms state-of-the-art methods in accuracy and robustness. Additionally, we report normative values for AVP volume in adult MR imaging, providing objective measurements for radiologists and ophthalmologists.

## 2 Materials and methods

### 2.1 Study population

This single-center study was approved by the institutional review board, with a waiver of informed consent due to its retrospective nature. Conducted in accordance with the latest version of the Declaration of Helsinki, the study evaluated patients with non-specific neurological symptoms reported between January and December 2022 in our institution’s picture archiving and communication system (PACS). Inclusion criteria were patients aged 18 to 60 who underwent brain 3D-T1WI during this period. Exclusion criteria included patients with intracranial or orbital tumors, craniocerebral or ocular trauma, deformity or infection, spherical refractive errors worse than −6D (18), or poor image quality.

The final cohort comprised 119 participants (61 females, 58 males; mean age 45.52 ± 12.18 years). Participants were randomly divided into training (74.8%, *n* = 89), validation (12.6%, *n* = 15), and test (12.6%, *n* = 15) cohorts.

### 2.2 MR examination

Images were acquired using a single 3.0T Siemens Skyra MRI scanner (Erlangen, Germany) with a 32-channel head coil. Patients were instructed to close their eyes and avoid eye movement during scanning. The 3D-T1WI sequence parameters were: TR = 2,000 ms, TE = 2.48 ms, flip angle = 8°, layer thickness = 1 mm, no gap, voxel size = 0.898 × 0.898 × 1 mm^3^, field of view = 20 cm × 20 cm, matrix size = 256 × 224, with a total of 192 layers.

### 2.3 Data annotations

**FIGURE 1:**
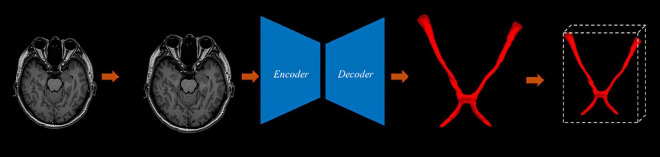
The proposed method encompasses data collection, preprocessing and labeling, deep learning predictions, result presentation, and volume calculation.

We converted the Digital Imaging and Communications in Medicine (DICOM) format T1-weighted images of the skull cross-section to the Neuroimaging Informatics Technology Initiative (NiFTI) format. To establish a reproducible, MRI-compatible landmark, we defined the specific range of the AVP based on prior anatomical knowledge (19, 20). This range extends from the optic nerve head to the lateral geniculate body—excluding the portion surrounded by cerebrospinal fluid—and encompasses the entire area of the bilateral optic nerves, optic chiasm, and bilateral optic tracts. Using ITK-SNAP 3.9.0 software,^1^ two neuroradiology trainees (Y.H., Q.L.) labeled each AVP layer by layer on the 3D-T1WI according to the defined anatomical landmarks, under the constant supervision of an experienced neuroradiologist (C.Z.). Accuracy was enhanced through iterative segmentation revisions until consensus with the senior neuroradiologist was reached. Figure 1 illustrates the overall scheme of the proposed method.

### 2.4 Segmentation model

**FIGURE 2:**
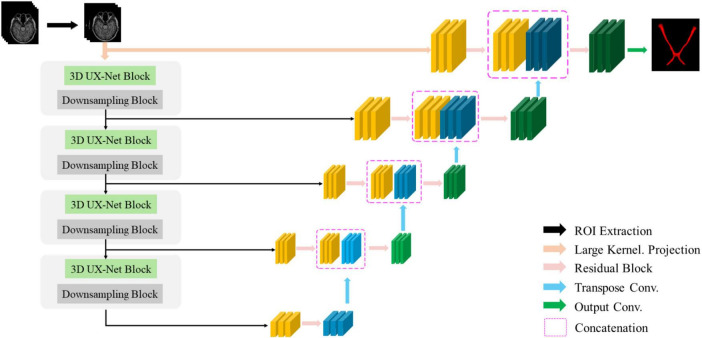
3D UX-Net employs a convolutional encoder backbone where large-kernel convolutions generate patch embeddings, with each stage’s downsampling block expanding channel-wise context.

We selected the 3D UX-Net as the segmentation network model for AVP because long-distance dependencies are crucial for accurately determining its absolute and relative positions. By analyzing the entire image’s structure and morphology, the network can swiftly identify and locate the AVP. Mainstream medical segmentation models like 3D U-Net have limited receptive fields and struggle to efficiently capture long-distance dependencies. In contrast, the 3D UX-Net, a lightweight volumetric convolutional network, expands the effective receptive field by using depth-wise convolutions with large kernel (LK) sizes (7 × 7 × 7). This approach enhances the network’s ability to capture long-distance spatial dependencies, which is essential for accurately segmenting structures rich in positional information that connect distant areas, such as nerve fibers. Additionally, the model’s performance is improved with fewer normalization and activation layers, reducing the number of parameters. Figure 2 provides an overview of 3D UX-Net, highlighting convolutional blocks as the encoder’s backbone. At the same time, the structures of the 3D U-Net, Swin UNETR, UNETR++, and Swin SMT models were constructed with reference to previous studies (14–17).

### 2.5 Training of the segmentation model

We utilized an NVIDIA GeForce RTX 3090Ti 24GB GPU, with software including Python 3.10, PyTorch 1.12.0, MONAI 1.0.0, OpenCV, NumPy, and SimpleITK. The AdamW optimizer and DiceCELoss function were employed for training. Image preprocessing parameters were set to a size of 16 × 448 × 256 (z, y, x), with automatic window width and level adjustments. A total of 119 datasets were randomly divided into training (*n* = 89), validation (*n* = 15), and test (*n* = 15) sets in an 8:1:1 ratio. The T1WI and the labeled files were input to segment the entire AVP. Training parameters included a batch size of 1, a learning rate of 0.0001, and 60 epochs, with validation performed after each training epoch.

### 2.6 Model evaluation

Evaluate the segmentation effectiveness using manually annotated labels as the benchmark for assessing the 3D UX-Net model’s performance on AVP. Evaluation metrics include the DSC, 95% Hausdorff distance (HD95), average symmetric surface distance (ASSD), and volume difference (VD) (21–25) (Equations 1–4). The calculation formulas are as follows:


(1) $$D\,S\,C\,\left(V_{\mathrm{gt}},V_{s\,e\,g}\right)=\frac{2\,\left|V_{\mathrm{gt}}\,⋂V_{s\,e\,g}\right|}{\left|V_{\mathrm{gt}}\right|+\left|V_{s\,e\,g}\right|},$$

Where *V*_gt_ represents the ground truth and *V_seg_* represents the automatic segmentation result.


(2) H⁢D⁢95⁢(A,B)=

$$p\,e\,r\,c\,e\,n\,t_{95}\,\left\{m\,a\,x\,\left\{s\,u\,p_{a\in A}\,i\,n\,f_{b\in B}\,d\,\left(a,b\right),s\,u\,p_{b\in B}\,i\,n\,f_{a\in A}\,d\,\left(b,a\right)\right\}\right\},$$

Where the *percent*_95_ refers to the 95th percentile, *A* and *B* are point sets where *A* is the predicted segmentation and *B* is the ground truth segmentation. *d* (*a*,*b*) measures the distance between point *a* and point *b*.


(3) $$A\,S\,S\,D\,\left(A,B\right)=\frac{1}{\left|A\right|+\left|B\right|}\,\left(\sum_{a\in A}\underset{b\in B}{m\,i\,n}d\,\left(a,b\right)+\sum_{b\in B}\underset{a\in A}{m\,i\,n}d\,\left(b,a\right)\right),$$

Where *A* < *B* are point sets representing the predicted segmentation and the true segmentation, respectively, and *d* (*a*, *b*) denotes the distance from point *a* to point *b*.


(4) $$V\,D=\frac{\left|T\,P_{s\,e\,g}-T\,P_{g\,t}\right|}{T\,P_{g\,t}},$$

Where *TP_seg_* is the true positive of the predicted segmentation and *TP_gt_* is the true positive of the true segmentation.

Data augmentation through geometric transformations (flipping) and photometric adjustments (brightness/contrast/hue variation) enhanced training diversity. We systematically evaluated test-set AVP segmentation accuracy across five architectures: 3D UX-Net, 3D U-Net, Swin UNETR, UNETR++, and Swin SMT. Computational complexity was quantitatively assessed via parameter counts, FLOPs, and inference time for all models. Furthermore, the AVP segmentation performance of the 3D UX-Net model was benchmarked against state-of-the-art segmentation outcomes documented in previous studies (9, 10, 26–28).

### 2.7 Volume measurement

We calculated the overall voxel volume (number of voxels × voxel size) from both the manually outlined and 3D UX-Net automatically segmented labels as the AVP volume, and then derived the average AVP volume for all participants based on the results of the 3D UX-Net automatic segmentation. Model comparison employed VD analysis, calculating absolute differences between automated and manual measurements across the test set.

### 2.8 Statistical analysis

Statistical analyses were performed using SPSS (v26.0) and Python (v3.10). A *P*-value less than 0.05 was considered statistically significant. Normal distribution of all continuous variables was examined using the *Shapiro-Wilk test*. Normally distributed variables were presented as mean ± standard deviation (SD), skewed distribution variables were described using median and interquartile range (IQR). Use *Levene’s test* to check if different groups have the same variance. If the data meets the criteria for normal distribution and homogeneity of variance, conduct a *one-way ANOVA*. If not, use the *Kruskal-Wallis test*. Post hoc tests were conducted using the *Dunn test*, with *Bonferroni correction* applied for multiple comparisons. The two-sided paired *Wilcoxon signed-rank test* was employed to compare AVP volumes between 3D UX-Net and manual segmentation predictions in the test cohort. The mean and standard deviation of AVP volumes were calculated to establish the 95% reference range for this population. When grouped by gender, statistical analysis was conducted using a *two-sample t*-test. For age groups differing by 10 years, the 95% reference intervals of AVP volume were calculated for each group, and trends were illustrated using a line chart.

## 3 Results

### 3.1 Segmentation results

The proposed 3D UX-Net achieved state-of-the-art DSC of 0.893 ± 0.017, outperforming the 3D U-Net (0.875 ± 0.019), Swin UNETR (0.870 ± 0.017), UNETR++ (0.861 ± 0.020), and Swin SMT (0.888 ± 0.017). Morphological analysis further confirmed its advantages, demonstrating the lowest ASSD of 0.234 mm [0.188–0.273], which significantly outperformed 3D U-Net (*p* = 0.003). All models exhibited identical median HD95 values of 1 mm (IQR = 1–1), indicating consistent control of extreme boundary errors across architectures. For surface distance accuracy, the 3D UX-Net achieved the lowest (0.234 mm [0.188–0.273]), significantly outperforming the 3D U-Net (0.404 mm [0.265–0.463], *p* = 0.003) and UNETR++ (0.338 mm [0.260–0.422], *p* = 0.021).

**FIGURE 3:**
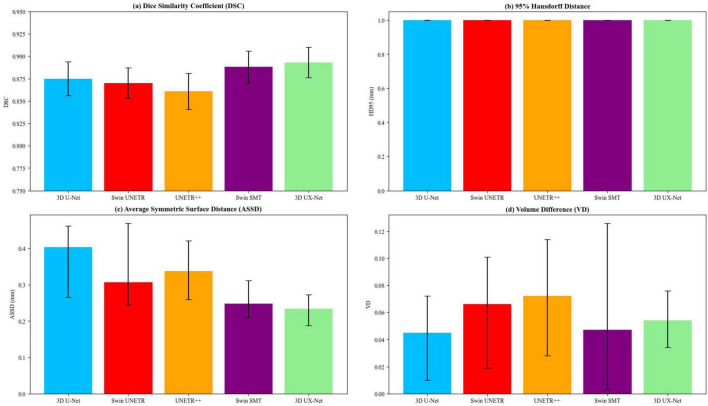
Comparative analysis of segmentation performance across 3D UX-Net, 3D U-Net, Swin UNETR, UNETR++, and Swin SMT on the test set, with quantitative evaluations including **(a)** Dice similarity coefficient, **(b)** 95% Hausdorff distance, **(c)** average symmetric surface distance, and **(d)** volume difference.

In terms of volume measurement, the Swin SMT exhibited the lowest median VD (0.047 [0.003–0.126]); however, its wider IQR suggested potential instability. No significant VD differences were observed between the 3D U-Net (0.045 [0.010–0.072]) and 3D UX-Net (0.054 [0.034–0.076], *p* = 0.27) (Table 1; Figure 3).

**TABLE 1:** Segmentation performance comparison of 3D UX-Net, 3D U-Net, Swin UNETR, UNETR++, and Swin SMT on the test set.

Model architecture	DSC (Mean ± SD)	HD95 (mm) (Median [IQR])	ASSD (mm) (Median [IQR])	VD (Median [IQR])
3D U-Net	0.875 ± 0.019	1 [1-1]	0.404 [0.265–0.463]	**0.045 [0.010–0.072]**
Swin UNETR	0.870 ± 0.017	1 [1-1]	0.307 [0.244–0.470]	0.066 [0.019–0.101]
UNETR++	0.861 ± 0.020	1 [1-1]	0.338 [0.260–0.422]	0.072 [0.028–0.114]
Swin SMT	0.888 ± 0.018	1 [1-1]	0.248 [0.210–0.312]	0.047 [0.003–0.126]
3D UX-Net	**0.893 ± 0.017**	1 [1-1]	**0.234 [0.188–0.273]**	0.054 [0.034–0.076]

Data are presented as mean ± SD or median [IQR]. SD, standard deviation; IQR, interquartile range; DSC, Dice similarity coefficient; HD95, Hausdorff distance 95%; ASSD, average symmetric surface distance; VD, volume difference. Bold values indicate the best performance in segmentation metrics (DSC, ASSD, VD).

**FIGURE 4:**
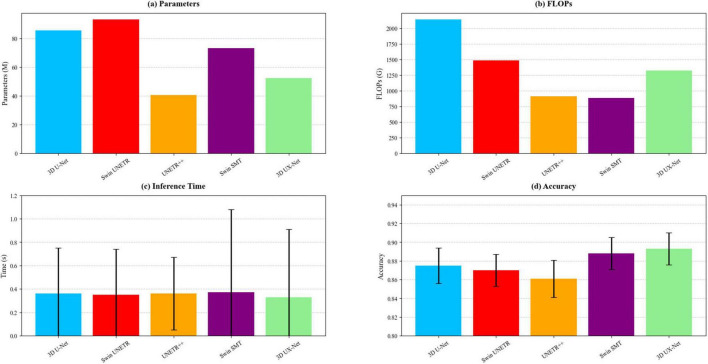
Comparison of computational complexity across the five models, including **(a)** number of parameters, **(b)** FLOPs, **(c)** inference time, and **(d)** accuracy.

The 3D UX-Net balances accuracy and efficiency with 52.39M parameters, a 38.6% reduction from 3D U-Net (85.43M), while outperforming Transformer-based Swin UNETR (92.96M) and Swin SMT (73.05M). Although UNETR++ has the fewest parameters (40.48M), its accuracy remains subpar (Table 1). In computational efficiency, 3D UX-Net (1325.73G FLOPs) reduces computational load by 38% versus 3D U-Net and is 10.7% more efficient than Swin UNETR. Swin SMT achieves the lowest FLOPs (886.03G) but shows higher accuracy variability. All models show comparable inference times, though 3D UX-Net and Swin SMT exhibit greater latency fluctuations than UNETR++. Table 2 and Figure 4 delineates the comparative analysis of computational complexity parameters across the five architectures.

**TABLE 2:** Cross-model comparison of computational complexity.

Model architecture	Parameters (M)	FLOPs (G)	Inference time (s)
3D U-Net	85.43	2139.34	0.36 ± 0.39
Swin UNETR	92.96	1485.11	0.35 ± 0.39
UNETR++	**40.48**	912.23	0.36 ± 0.31
Swin SMT	73.05	**886.03**	0.37 ± 0.71
3D UX-Net	52.39	1325.73	**0.33 ± 0.58**

Results were presented as mean ± SD. SD, standard deviation; FLOPs, floating point operations per second. Bold values indicate the best performance in lightweight metrics (Parameters, FLOPs, Inference Time).

**FIGURE 5:**
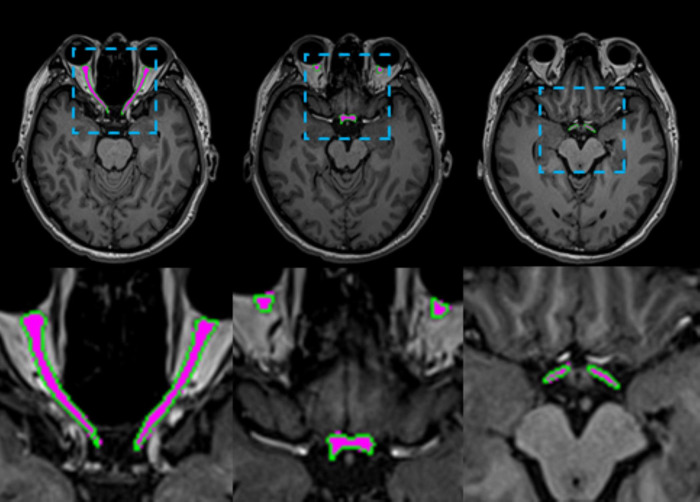
Visual comparisons of AVP segmentation examples are shown. The predicted AVP contours are overlaid in green on a representative axial T1-weighted MRI of the brain from one test subject, with manual annotations overlaid in magenta for reference. The inset provides a close-up comparison of the predicted values.

The 3D UX-Net model exhibited minimal volumetric discrepancy in AVP predictions (1514.56 ± 165.52 mm^3^) compared to ground truth annotations, with detailed per-subject performance metrics documented in Table 3. As illustrated in Figure 5, automated contours generated by 3D UX-Net showed high anatomical concordance with manual delineations, achieving submillimeter boundary accuracy (0.23 mm) that meets precision requirements for image-guided surgical interventions.

**TABLE 3:** The 3D UX-Net model performance for AVP segmentation on the test subjects.

Subjects	DSC	HD95 (mm)	ASSD (mm)	VD	Volume (mm^3^)
HC001	0.894	1.0	0.237	0.054	1414.44
HC002	0.886	1.0	0.273	0.076	1504.94
HC003	0.915	1.0	0.159	0.034	1417.05
HC004	0.917	1.0	0.176	0.007	1777.66
HC005	0.867	1.41	0.428	0.093	1566.76
HC006	0.906	1.0	0.183	0.037	1351.95
HC007	0.896	1.0	0.263	0.061	1877.91
HC084	0.899	1.0	0.204	0.020	1723.63
HC085	0.897	1.0	0.219	0.035	1505.58
HC086	0.880	1.0	0.229	0.054	1405.34
HC087	0.895	1.0	0.259	0.067	1594.11
HC088	0.897	1.0	0.235	0.045	1428.13
HC089	0.913	1.0	0.188	0.007	1438.54
HC132	0.860	1.0	0.346	0.241	1419.66
HC133	0.872	1.0	0.548	0.182	1292.73
Mean/median	**0.893 ± 0.017**	**1 [1**-**1]**	**0.234 [0.188–0.273]**	**0.054 [0.034–0.076]**	**1514.56 ± 165.52**

Within-group statistical data are shown in bold and presented as mean ± SD or median [IQR]. SD, standard deviation; IQR, interquartile range; DSC, Dice similarity coefficient; HD95, Hausdorff distance 95%; ASSD, average symmetric surface distance; VD, volume difference; HC, healthy control.

Comparative analysis with prior studies addressing analogous AVP segmentation tasks revealed that the 3D UX-Net architecture achieves statistically significant improvements in segmentation accuracy, as empirically demonstrated in Table 4.

**TABLE 4:** Comparison of our method with the AVP segmentation methods reported in the literature.

Methods	Year	No. of subjects	MRI sequence	Voxel size (mm^3^)	DSC	HD95 (mm)	ASSD (mm)
3D FCN + SPDM. (Zhao et al.)	2019	93	T1w	1 × 1 × 1	0.85 ± 0.01	4.64 ± 1.41	0.37 ± 0.04
3D U-Net (Ai et al.)	2020	93	T1w	1 × 1 × 1	0.86 ± 0.01	3.56 ± 1.89	0.34 ± 0.05
TPSN (Li et al.)	2021	102	T1w + FA	1.25 × 1.25 × 1.25	0.85 ± 0.02	2.33	0.16
3D U-Net (van Elst et al.)	2022	40	T2w	0.25 × 0.25 × 0.7/ 0.27 × 0.27 × 0.3	0.84	0.64	0.14
CNTSeg (Xie et al.)	2023	102	T1w + FA + fODF peaks	1.25 × 1.25 × 1.25	0.82 ± 0.02	–	–
3D UX-Net (ours)	**–**	**119**	**T1w**	**0.90 × 0.90 × 1**	**0.893 ± 0.017**	**1 [1** *-* **1]**	**0.234 [0.188–0.273]**

Data are presented as mean ± SD or median [IQR]. SD, standard deviation; IQR, interquartile range; DSC, Dice similarity coefficient; HD95, Hausdorff distance 95%; ASSD, average symmetric surface distance; FCN, fully convolutional network; SPDM, spatial probabilistic distribution map; CNTSeg, cranial nerves tract segmentation; FA, fractional anisotropy; fODF, fiber orientation distribution function; TPSN, two parallel stages network. Bold values indicate the best performance in AVP segmentation.

### 3.2 Volume measurement results

**FIGURE 6:**
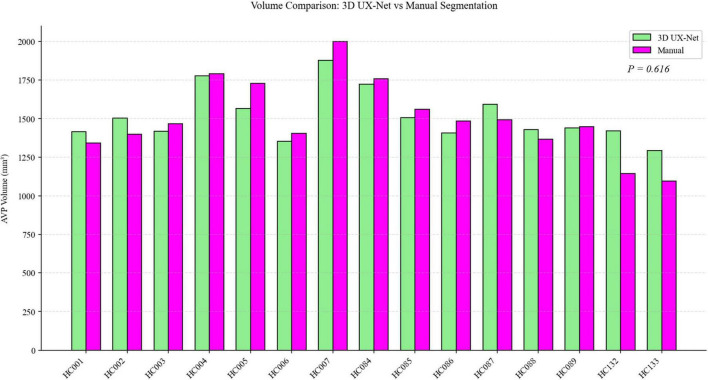
Comparison of AVP volumes measured by 3D UX-Net and manual segmentation on the test set.

In the test set, the detailed results of applying the 3D UX-Net model to calculate individual AVP volumes are presented in Table 3. The average volume of the 3D UX-Net automatically segmented AVP across all subjects was 1446.78 ± 245.62 mm^3^, with an average VD of 0.068 ± 0.064 compared to manual labeling. A *2-tailed Wilcoxon signed-rank test* showed no significant difference (*p* = 0.616) in AVP volumes predicted by 3D UX-Net and manual segmentation on the test cohort. The volumes measured by 3D UX-Net and manual methods are visualized in Figure 6.

### 3.3 Volume comparison

**FIGURE 7:**
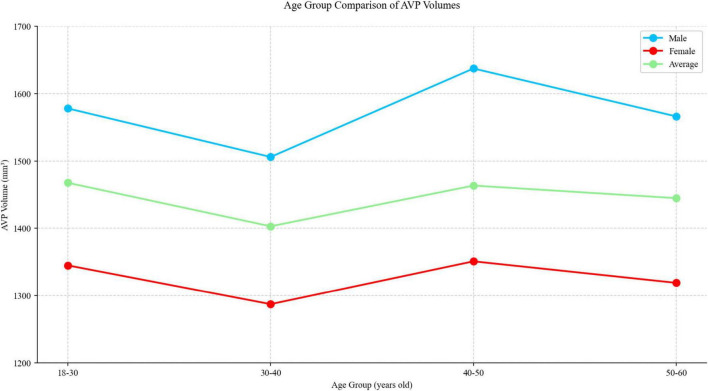
Comparison of AVP volumes across various gender and age groups using manual segmentation results for all subjects.

We calculated the AVP volume for all subjects using the automatic segmentation results from 3D UX-Net and compared the volumes between different sex and age groups. Male subjects exhibited significantly larger AVP volumes than females (Males: 1572.33 ± 242.90 mm^3^ vs. Females: 1327.41 ± 181.29 mm^3^; *p* < 0.001), despite comparable age distributions between groups (*p* = 0.63). No significant association emerged between age and AVP volume across the studied cohort. The complete statistical breakdown is visualized in Figure 7 and tabulated in Table 5.

**TABLE 5:** Volume distribution of the anterior visual pathway across different age groups.

Age (years old)	Gender	Number	Volume (mean ± SD mm^3^)	95%IC (mm^3^)	*P*-value
18-30	Male	10	1578.03 ± 278.51	(1378.80, 1777.26)	0.035*
	Female	9	1344.80 ± 136.44	(1239.92, 1449.68)	
	Total	19	1467.55 ± 56.83	(1348.15, 1586.96)	
30-40	Male	9	1505.87 ± 297.14	(1277.47, 1734.27)	0.079
	Female	8	1287.03 ± 164.16	(1149.79, 1424.27)	
	Total	17	1402.89 ± 63.53	(1268.21, 1537.56)	
40-50	Male	11	1637.36 ± 186.11	(1512.33, 1762.39)	0.001*
	Female	17	1350.85 ± 189.35	(1248.87, 1452.83)	
	Total	28	1463.41 ± 44.90	(1371.28, 1555.54)	
50-60	Male	28	1566.12 ± 237.63	(1473.97, 1658.26)	0.000*
	Female	27	1318.82 ± 193.94	(1242.10, 1395.54)	
	Total	55	1444.71 ± 33.55	(1377.45, 1511.98)	

*The differences are statistically significant.

## 4 Discussion

In this study, we developed and validated an automated framework for AVP segmentation and volume measurement in brain 3D T1WI. The 3D UX-Net accurately, robustly, and quickly detects AVP contours and quantifies volumes, demonstrating a higher mean DSC than the 0.82 reported in Xie et al. (10). Compared to the 3D U-Net, Swin UNETR, UNETR++, and Swin SMT, the 3D UX-Net emerged as the most robust architecture, achieving state-of-the-art DSC and ASSD while maintaining competitive volumetric consistency. Despite comparable HD95 values across all models, 3D UX-Net’s superior surface distance accuracy and stability suggest enhanced capability for fine-grained anatomical segmentation. These results highlight the importance of balancing spatial precision with volumetric reliability in medical image analysis. Our method’s reliability was confirmed in an independent cohort and was compared to manual segmentation, which is labor-intensive and impractical in busy clinical settings. Our approach offers rapid predictions with accuracy comparable to manual results, providing timely AVP contour and volume data for radiologists. Additionally, we offer a reference range for adult AVP volume, useful for diagnosing AVP-related diseases. We also analyzed AVP volume variations across different age groups and genders using manual segmentation.

Variability in AVP measurements is likely influenced by factors such as MRI sequences, slice thickness, measurement locations, and measurement methods. While some researchers prefer T1-weighted images for visualizing the optic nerve (29, 30), others advocate for standard or novel T2-weighted sequences that may better distinguish the optic nerve from cerebrospinal fluid (31, 32). We chose to segment high-resolution T1WI due to their near isotropic voxel size, avoiding image resolution degradation during multiplanar reconstruction. Additionally, this sequence is included in our institution’s standard radiological acquisition. We anticipate no significant differences in AVP measurements using 3D-T1WI sequences across centers employing MRI systems from different manufacturers.

Previously, studies have segmented orbital structures in CT images using semi-automatic or automatic methods for diagnostic imaging and surgical planning. Pravatà et al. (11) proposed a deep learning-guided partitioned shape model for anterior visual pathway segmentation, initially utilizing a marginal space deep-learning stacked autoencoder to locate the pathway, then combining a novel partitioned shape model with an appearance model to guide segmentation (6). Zhao et al. (9) introduced a method for visual pathway segmentation in MRI based on a 3D FCN combined with a Spatial Probabilistic Distribution Map (SPDM), which represents the probability that a voxel belongs to a specific tissue by summing all manual labels in the training dataset. Incorporating SPDM effectively overcomes issues of low contrast and blurry boundaries, achieving improved segmentation performance. Harrigan et al. (33) enhanced the multi-atlas segmentation method by introducing a technique for quantitatively measuring the optic nerve and cerebrospinal fluid sheath, demonstrating its potential to differentiate patients with optic nerve atrophy or hypertrophy from healthy individuals. Aghdasi et al. (34) defined a volume of interest (VOI) encompassing the desired structures using anatomical prior knowledge, employing it for rapid localization and effective segmentation of orbital structures—globes, optic nerves, and extraocular muscles—in CT images. This method is precise, efficient, requires no training data, and its intuitive pipeline enables adaptation to other structures. In our study, we used the 3D UX-Net network structure to train the segmentation model in two steps using a coarse-to-fine method. Compared to previous studies on similar AVP segmentation tasks, the 3D UX-Net architecture significantly improves segmentation accuracy. Additionally, we observed that all models had a median HD95 of 1 mm with an IQR of [1-1], showing only a small number of outliers around 1.4 mm. Possible explanations include: a. All subjects were free of optic nerve-related disorders, and anatomical variations in the AVP are smaller in healthy states compared to pathological conditions; b. While most AVP boundaries are distinct due to high tissue contrast, partial volume effects from adjacent structures in rare cases may contribute to elevated HD95 values.

Comparative analysis of computational complexity among the 3D U-Net, Swin UNETR, UNETR++, Swin SMT, and 3D UX-Net reveals that 3D UX-Net achieves the optimal accuracy-efficiency balance in parameter efficiency and computational precision, providing a viable solution for deploying high-accuracy real-time segmentation systems in clinical settings. However, its FLOPs still lag significantly behind Swin SMT, revealing potential for further compression of computational redundancy.

Several studies have shown that age and ethnicity influence optic nerve size (35, 36). However, our findings revealed no clear correlation between age and optic nerve volume, possibly due to variations such as different examination techniques and post-processing programs. Inglese et al. (37) included controls with a mean age of 37 years old from an Italian population, whereas our study’s controls had an older mean age of 46 years old from an Asian population. We believe these factors may also affect optic nerve volume, which requires verification through an expanded sample size.

Mncube and Goodier (38) showed that the transverse diameter of the optic chiasm is the most reliable measure of the AVP in adults because it is the largest structure and easiest to measure. However, due to the irregular morphology of the AVP, measurements of the optic chiasm may not capture all its features. Our comprehensive volumetric assessment addressed this limitation. In our test set, two subjects exhibited lower segmentation accuracy and significantly greater volume differences than others. This may be attributed to the tortuous course of the optic nerve within the orbital segment of these subjects, along with a longer segment parallel to the ophthalmic artery (39).

This study has several limitations. Firstly, it relies on single-center data, necessitating future validation in broader clinical settings with larger datasets. Secondly, our training cohort included only subjects with normal AVP images, excluding related diseases such as congenital malformations, inflammation, and tumors. We intend to apply this method for automated segmentation and volume measurement of AVP and for differential diagnosis of inflammatory demyelinating diseases. Lastly, we only used the 3D-T1WI sequence to measure the AVP’s total volume. Future research could include T2-FLAIR and DTI sequences to improve accuracy and reliability. Additionally, refining measurement metrics such as segmented volume, length, and cross-sectional area could provide more comprehensive evaluation data.

## 5 Conclusion

In this study, we introduced the 3D UX-Net for segmenting AVP on brain 3D-T1WI, which outperformed the 3D U-Net, Swin UNETR, UNETR++, and Swin SMT. Our convolutional neural network, trained and validated on MRI scans from 119 healthy individuals, achieved an average DSC of 0.893 for AVP segmentation with a volume error under 1%. Using the AVP volume from the normal study group as a benchmark, we can quantitatively evaluate the volume difference between a given MR image and the baseline, automatically identifying abnormalities when differences exceed the normal distribution. This comparison provides radiologists with valuable information within a clinically practical timeframe, aiding in the detection of subtle AVP lesions. The approach’s high accuracy and computational efficiency make it suitable for quantifying and distinguishing optic nerve-related diseases.

## Data Availability

The original contributions presented in the study are included in the article/supplementary material, further inquiries can be directed to the corresponding authors.

## Footnotes

1
http://www.itksnap.org/pmwiki/pmwiki.php
